# Ganglioside GM1 Targets Astrocytes to Stimulate Cerebral Energy Metabolism

**DOI:** 10.3389/fphar.2021.653842

**Published:** 2021-04-28

**Authors:** Charles Finsterwald, Sara Dias, Pierre J. Magistretti, Sylvain Lengacher

**Affiliations:** GliaPharm SA, Geneva, Switzerland

**Keywords:** ganglioside, GM1, astrocyte, lactate, metabolism, glycolysis

## Abstract

Gangliosides are major constituents of the plasma membrane and are known to promote a number of physiological actions in the brain, including synaptic plasticity and neuroprotection. In particular, the ganglioside GM1 was found to have a wide range of preclinical and clinical benefits in brain diseases such as spinal cord injury, Huntington’s disease and Parkinson’s disease. However, little is known about the underlying cellular and molecular mechanisms of GM1 in the brain. In the present study, we show that GM1 exerts its actions through the promotion of glycolysis in astrocytes, which leads to glucose uptake and lactate release by these cells. In astrocytes, GM1 stimulates the expression of several genes involved in the regulation of glucose metabolism. GM1 also enhances neuronal mitochondrial activity and triggers the expression of neuroprotection genes when neurons are cultured in the presence of astrocytes. Finally, GM1 leads to a neuroprotective effect in astrocyte-neuron co-culture. Together, these data identify a previously unrecognized mechanism mediated by astrocytes by which GM1 exerts its metabolic and neuroprotective effects.

## Introduction

Gangliosides are sialic acid-containing glycosphingolipids, and are naturally present in the plasma membrane of most vertebrate cells, particularly in the brain where they constitute up to 10% of the total lipid mass. Gangliosides play important roles in many physiological processes including cell differentiation, apoptosis, neuronal protection and neurotrophins release (Magistretti et al., 2019). Changes in the relative abundance of specific gangliosides were reported in neurodegenerative disease, including Alzheimer’s disease (AD) (Blennow et al., 1991; Blennow et al., 1992), Parkinson’s disease (PD) (Wu et al., 2012), Huntington’s disease (HD) (Desplats et al., 2007; Maglione et al., 2010), multiple sclerosis (MS) (Miyatani et al., 1990; Zaprianova et al., 2001) and amyotrophic lateral sclerosis (ALS) (Dodge et al., 2015). Specifically, deficiency in the expression of GM1, one of the predominant brain gangliosides, has been reported in neurological diseases such as PD (Wu et al., 2012), HD (Maglione et al., 2010) and traumatic brain injury (TBI) (Mahadik et al., 1988; Rubovitch et al., 2017).

Different studies have highlighted the important neuroprotective and neurotrophic actions of GM1 *in vitro*, in animal studies and in clinical trials, demonstrating the potential therapeutic effect of GM1 to slow or even reverse the progression of neurological diseases such as HD (Di Pardo et al., 2012; Alpaugh et al., 2017), stroke (Lenzi et al., 1994), spinal cord injury (SCI) (Geisler et al., 1991), AD (Yang et al., 2013) and PD (Schneider et al., 1998). Genetic defects that affect ganglioside synthesis were also found to result in severe early onset neurological diseases (Li & Schnaar, 2018). A number of pathologies with GM1 deficiency are also linked to deficits in brain energy metabolism. For instance, HD is characterized by a failure in brain energy metabolism, as shown by positron emission tomography (PET) studies (Kuwert et al., 1990), while lactate metabolism is altered in R6/2 and HdhQ7/111 mouse models of HD (Solis-Maldonado et al., 2018). In addition, astrocytic glycolysis and lactate are reduced in aging (Harris et al., 2016; Goyal et al., 2017), AD (Zhang et al., 2018) and ALS (Lee et al., 2012).

GM1 is not only present in neuronal membranes in the brain, but also in other cell types including astrocytes (Kim et al., 1986; Facci et al., 1987). Astrocytes are essential cellular players in the regulation of numerous brain physiological mechanisms, such as neurovascular coupling, regulation of brain energy metabolism, storage of glycogen and neuroinflammation. Aerobic glycolysis is triggered in astrocytes upon glutamatergic transmission, which leads to lactate secretion (Pellerin and Magistretti, 1994). After transfer from astrocytes to neurons, lactate is transformed into pyruvate to enter neuronal tricarboxylic acid (TCA) cycle through the astrocyte-neuron lactate shuttle (ANLS) (Machler et al., 2016; Magistretti and Allaman, 2018). This cellular cerebral dialogue not only provides energy, but also acts as an NADH-mediated redox signal in neurons since NADH is formed upon conversion of lactate to pyruvate. A number of studies underlined the essential role of astrocyte-mediated lactate production in neuroprotection (Berthet et al., 2012; Jourdain et al., 2018), memory formation (Newman et al., 2011; Suzuki et al., 2011; Harris et al., 2019) and transcription of neuronal plasticity genes (Yang et al., 2014; Margineanu et al., 2018). Treatment with gangliosides was shown to affect astrocytic morphology, growth and activation, supporting the notion that astrocytes are responsive to gangliosides and may be mediators of GM1 effects (Hefti et al., 1985; Skaper et al., 1986; Facci et al., 1987; Pyo et al., 1999; Min et al., 2004; Jou et al., 2006; Lee et al., 2012).

In this study, we analyzed the effect of GM1 on astrocytes and neuronal metabolism in monocultures of each cell type, and in co-cultures. We showed that GM1 modulates astrocytic glycolysis, leading to glucose uptake, mobilization of glycogen and secretion of lactate, as well as the expression of glucose metabolism genes in astrocytes. Further, we found that GM1 enhances mitochondrial activity and triggers the expression of neuroprotection genes in neurons through mechanisms that require the presence of astrocytes.

## Materials and Methods

### Animal Experimentation

All experiments were carried out in accordance with the Swiss Federal Guidelines for Animal Experimentation and were approved by the Cantonal Veterinary Office for Animal Experimentation (Geneva, Switzerland).

### Primary Mouse Cortical Astrocytes

Primary cultures of cortical astrocytes were obtained from post-natal day 1 (P1) OF1 mice (Charles River Laboratories), as previously described (Allaman et al., 2010). Cells were plated at an average density of 10^5^ cells/cm^2^ on poly-L-ornithine-coated (100 mg/L) 96, 12 or 6-well culture plates and incubated at 37°C in a humidified atmosphere containing 5% CO_2_/95% air. Culture medium consisted in Dulbecco’s Modified Eagle’s Medium (DMEM) with 25 mM glucose (Sigma Aldrich), supplemented with 44 mM bicarbonate and 10% fetal calf serum (FCS, Bioconcept). Culture medium was renewed twice a week. Experiments were performed on confluent cell cultures at 15–16 days *in vitro*.

### Primary Mouse Cortical Neurons

Primary cultures of cortical neurons were prepared from embryonic day 17 (E17) OF1 mouse embryos (Charles River Laboratories), as previously described (Finsterwald et al., 2010). Cells were plated at an average density of 1.5 × 10^5^ cells/cm^2^ on poly-D-lysine-coated (100 mg/L) 12-well plates. Cells were maintained at 37°C in a humidified atmosphere containing 5% CO_2_ and 95% air, and were used at 14 days *in vitro*. Neuronal culture medium consisted in Neurobasal medium (Invitrogen) supplemented with B27 (Invitrogen) and GlutaMAX (Invitrogen).

### Primary Mouse Cortical Astrocyte-Neuron Co-cultures

Primary cultures of cortical neurons and astrocytes were prepared as described above, except that the astrocytes were grown at an average density of 10^5^ cells/cm^2^ on 15 mm diameter Nunc Thermanox coverslips (Thermo Fisher) with two 3-mm paraffin beads on each of them, placed in 12-well plate and coated overnight with poly-L-ornithine (100 mg/L). At the day of experiment, the co-culture was initiated by transferring the coverslips in each well of 12-well plates neuronal cultures, with astrocytes on coverslips facing neurons in the well, being separated by 3 mm paraffin beads.

### Cell Treatment

Cells were treated with ganglioside GM1 provided by TRB Chemedica International SA (Geneva, Switzerland), which was solubilized in 100% dimethylsulfoxide (DMSO). Concentrations of GM1 tested ranged from 6 to 50 μM in a final volume of 0.1% DMSO, identical to Vehicle (0.1% DMSO), for a duration of 30 min to 6 h depending on the experiment. Stimulation of primary astrocytes was performed in medium composed of DMEM supplemented with 5 mM Glucose and 44 mM bicarbonate (pH 7.2). Astrocyte culture medium was replaced with astrocyte stimulation medium at least 1 h before stimulation. Neuronal culture medium was not changed before stimulation of primary neurons or primary astrocyte-neuron co-cultures.

### Lactate Secretion Quantification

L-lactate concentration was determined by enzymatic method, as described previously (Allaman et al., 2010). Briefly, samples of culture medium were taken up after treatment, and lactate concentration in the medium was quantified with Lactate Dehydrogenase (LDH)-based assay. The analysis consisted in the measurement of lactate conversion to pyruvate, which is catalyzed by LDH, and results into the reduction of NAD^+^ into NADH. NADH fluorescence was quantified using Tecan fluorescence microplate reader (excitation 340 nm, emission 440 nm). Concentrations of L-lactate were determined using L-lactate standard curve.

### Glycogen Quantification

Intracellular glycogen was measured through NADPH formation due to glycogen mobilization. After stimulation, cells were washed with ice-cold PBS and lyzed by sonication in 600 µL of 30 mM Tris HCl. After an incubation period of 30 min at 90°C, 28 μL of a 0.1 M acetate buffer (pH 4.6) was added to 250 μL-aliquots, which were then separated in two; 5 μL amyloglucosidase was added or not to each aliquot, which were incubated for 2 h at 37°C. After centrifugation, 150 μL of a 0.1 M Tris-HCl buffer (pH 8.1) containing 3.3 mM MgCl_2_, 0.67 mM ATP, 0.67 mM NADP and 1.87% hexokinase/glucose-6-phosphate dehydrogenase (Roche) was added to 20 μL supernatant. Next, fluorescence proportional to NADPH concentrations was read using Tecan fluorescence microplate reader (340 nm excitation, 440 nm emission). Glycogen concentration was calculated by subtracting glucose value of samples that contained amyloglucosidase to samples that did not contain it. Concentrations of glucose units were determined from standard curve. Glycogen content was normalized to protein content, which was measured with micro BCA Protein Assay kit (Thermo Scientific), as described in manufacturer’s instructions.

### Oxidoreductase Thiazol Blue Tetrazolium Bromide (MTT) Assay

Mitochondrial activity was measured using the MTT reduction assay. Astrocytes grown in 96-well plates (astrocyte cultures) or on coverslips (astrocytes in co-cultures), and neurons grown in 12-well plates were used for MTT assays. 2 h before the end of the stimulation, MTT (Sigma Aldrich) was directly added into the medium to a final concentration of 0.5 mg/ml. Cells were then incubated for 2 h. After incubation, supernatant was removed, cells were washed with PBS and 50 µL or 500 µL DMSO was added in each well (for 96-well or 12-well plates, respectively) to stop the reaction and solubilize reduced MTT (formazan), which amount is proportional to the activity of oxidoreductase enzymes. Formazan was quantified using Tecan absorbance microplate reader at the absorbance of 570 nm.

### Inner Mitochondrial Potential (Δψm) Determination

A solution containing 6.25 μg/ml JC-1 dye (Invitrogen) was added to each well of 12-well culture plates 15 min before the end of stimulation. JC-1 dye exhibits mitochondrial potential-dependent fluorescence (Reers et al., 1991). At low potential, monomers are formed and display green fluorescence while at high potential, JC-1 aggregates and displays red fluorescence. After incubation, fluorescence was measured using Tecan fluorescence microplate reader (594 nm emission/497 nm excitation (red), and 527 nm emission/497 nm excitation (green). The mitochondrial potential level was quantified by calculating the ratio of both fluorescence values.

### Hydrogen Peroxide (H2O2) Quantification

Hydrogen peroxide (H_2_O_2_) accumulated in cell culture medium was detected enzymatically with Amplex red H_2_O_2_ probe (Invitrogen). Astrocytes grown in 96-well plates were treated for 6 h in stimulation medium complemented with 10 μM Amplex red and 1 U/ml HRP (Sigma Aldrich). Oxidation of Amplex red is catalyzed by the horseradish peroxidase (HRP) in presence of H_2_O_2_, which results in fluorescent resorufin that was quantified using Tecan fluorescence microplate reader (excitation 545 nm, emission 590 nm).

### ATP/ADP Quantification

Intracellular ATP content was measured enzymatically in a luciferin/luciferase assay. ATP was determined with the CellTiter-Glo Luminescent Cell Viability Assay (Promega). In presence of ATP, Mg^2+^ and O_2_, luciferin is oxygenated by luciferase into oxyluciferin, which emits light that is proportional to ATP concentrations. At the end of the stimulation, culture medium was removed and 400 μL lysis buffer, which consisted of Tricine buffer solution (40 mM Tricine, 3 mM EDTA, 85 mM NaCl, 3.6 mM KCl, 100 mM NaF and 0.1% saponin, pH 7.4), was added to each well of 12-well culture plates. Each sample was separated in two 30 μL aliquots: one for ATP and the one for ATP + ADP quantification. For the ATP + ADP, 10 μL of converting solution (100 mM Tricine, 100 mM MgSO_4_, 25 mM KCl, 1 mM phosphoenolpyruvate and 100 U/ml pyruvate kinase, pH 7.75) was added to each aliquot, while the same solution without phosphoenolpyruvate and pyruvate kinase was added to the samples for ATP measurement. 10 μL of MgCl_2_ solution (4 mM tricine and 100 mM MgCl_2_) was added to each sample and incubated for 5 min at 37°C. Finally, 30 μL of CellTiter-Glo reagent (Promega) was added and luminescence was detected using Tecan luminescence microplate reader at intervals of 1 min until plateau was reached. ADP values were calculated by subtracting ATP values from ATP + ADP values. Concentrations were determined from ATP and ADP standard curves.

### NADH/NAD^+^ Quantification

Intracellular NADH/NAD^+^ redox ratio was measured using enzymatic quantification, as described elsewhere (Zerez et al., 1987). Briefly, cells were washed in PBS after treatment and 600 μL of 20 mM NaHCO_3_/100 mM Na_2_CO_3_ buffer (pH10) containing 1 M nicotinamide was added followed by flash freeze to disrupt cell membranes. Once thawed, samples were separated in two parts: one for the dosage of NADH + NAD^+^ and the other one for NADH only. Samples for NADH detection were heated at 60°C for 30 min to destroy NAD^+^. Next, 150 μL of a reaction mix composed of 133 mM bicine, 5.33 mM EDTA, 0.56 mM MTT, 2.21 mM PES, 667 mM ethanol and 40 U/ml alcohol dehydrogenase was added to each of the 50 μL samples. Reduction of MTT was followed by measuring absorbance at 570 mm using Tecan fluorescence microplate reader, and NAD^+^ values were calculated by subtracting NADH from NADH + NAD^+^ values.

### Quantitative Polymerase Chain Reaction (qPCR)

RNA extraction was performed with the NucleoSpin RNA Plus kit (Macherey-Nagel), as indicated in the manufacturer’s instruction. Reverse transcription was done in a 20-μL reaction using 100 ng of RNA, with the High Capacity RNA-to-cDNA Kit (Life Technologies) as indicated in the manufacturer’s instruction. Quantitative determination of mRNA of metabolic genes and of synaptic plasticity and neuroprotection genes was performed with the QuantStudio 6 Flex Real-Time PCR System (Applied Biosystems), using the Power UP SYBR Green Master mix (Applied Biosystems) as a Taq polymerase master mix. The following primers were used: *Arc*, forward 5′-CCA GTC TTG GGC AGC ATA GC-3′, reverse 5′-TCT GCT CTT CAC TGG TAT GAA TC-3’; *Bdnf,* forward 5′-CCA TAA GGA CGC GGA CTT GT-3′, reverse 5′-GAG GCT CCA AAG GCA CTT GA-3’; *β-actin*, forward 5′-GCT TCT TTG CAG CTC CTT CGT-3′, reverse 5′-ATA TCG TCA TCC ATG GCG AAC-3’; *c-fos*, forward 5′-CGG AGG GAG CTG ACA-3′, reverse 5′-CTG CAA CGC AGA CTT CTC ATC T-3’; *Cyclophilin A,* forward 5′-CAA ATG CTG GAC CAA ACA CAA-3′, reverse 5′-GCC ATC CAG CCA TTC AGT CT-3’; *Egr1 (Zif268)*, forward 5′-GCC GAG CGA ACA ACC CTA-3′, reverse 5′-TTC AGA GCG ATG TCA GAA AAG-3’; *Egr4*, forward 5′-CTT GCT GCC GGA CCT CTA CT-3′, reverse 5′-AAA ACG CCT CCG GAA AGG-3’; *Eno2*, forward 5′-TGA TCT TGT CGT CGG ACT GTG T-3′, reverse 5′-GCC AGA CGT TCA GAT CTG CAT-3’; *Glut1,* forward 5′-CCA GCT GGG AAT CGT T-3′ reverse 5′-CTG CAT TGC CCA TGA TGG A-3’; *Hk*, forward 5′-AGG ACA TCA TGC GGG GCA GT-3′, reverse 5′-GTT GGC CAG GCA TTC GGC AA-3’; *Ldha,* forward 5′-TTG TCT CCA GCA AAG ACT GTG T-3′, reverse 5′-TTT CGC TGG ACC AGG TTG AG-3’; *Na+/K + ATPase α2 subunit,* forward 5′-ACC TGT GGC AAT CAC AAT GC-3′, reverse 5′-ACC TGT GGC AAT CAC AAT GC-3’; *Nr4a3,* forward 5′-CCA TCG CCA CCC AAT AGG-3′, reverse 5′-CGC ACA CGG CAC ATG TG-3’; *Pdh*, forward 5′-CCA TGG ACA CAG CAT GAG TGA-3′, reverse 5′-CAC CAT TCT ATC CTT GAG AAG CAT AA-3’; *Ptg* forward 5′-TGC​CTC​TCG​GTC​CAA​TGA​G-3′, reverse 5′-GGC ATG ACG GAA CTT GTC AA-3’;*Taldo*, forward 5′-GGA AAG GAG CTG GAG GAA CAG-3′, reverse 5′-CTG GGC GAA GGA GAA AAG C-3’.

All primers pairs were designed to overlap exon-exon junctions in order to avoid contamination signal from eventual genomic DNA. Samples were analyzed in duplicates. Relative gene expression was quantified using the comparative ΔΔ Ct method and normalized to *β-actin* and *cyclophilin A* reference transcript levels (Livak and Schmittgen, 2001).

### Neuroprotection Assay

Neuronal survival was determined by MTT in astrocyte-neuron co-cultures or neuronal monocultures that were exposed for 2 h to Veh. (0.1% DMSO) or GM1, followed by glutamate excitotoxic shock (100 μM for 10 min). After glutamate shock, medium was replaced with fresh stimulation medium without glutamate, which contained Veh or GM1 for 4 h. MTT was added to the medium 2 h before end of treatment and formation of formazan was quantified in neurons, as described above.

### Statistical Analyses

Statistical significance was calculated using unpaired bilateral Student’s t-tests, or by one-way analysis of variance (ANOVA) followed by Dunnett’s or Bonferroni’s *post-hoc* tests, using GraphPad Prism v.9.0. *, ** and *** refer to *p* values of <0.05, 0.01, and 0.001, respectively. All experiments are the average +standard error of the mean (S.E.M). of at least 3 independent experiments. Total number of replicates (n) is indicated in the figure legends.

## Results

**FIGURE 1:**
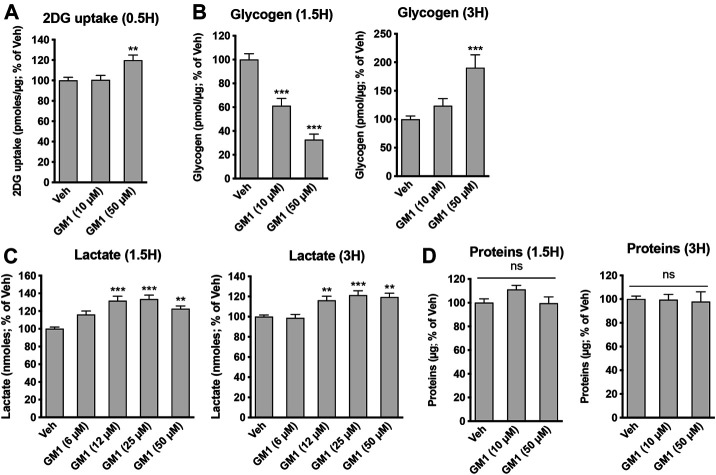
Modulation of astrocytic glycolysis by GM1. **(A)** 2-deoxyglucose (2DG) uptake by astrocytes during 30 min-treatment with Veh (DMSO 0.1%) or GM1 (10 and 50 μM). Data are shown as the average +SEM of 2DG uptake (pmoles/μg) expressed as % of Veh (*n* = 20). **(B)** Intracellular levels of glycogen in primary astrocytes after 1.5- and 3 h treatment with Veh (DMSO 0.1%) or GM1 (10 and 50 μM). Data are shown as the average +SEM of glycogen levels (pmoles/μg) expressed as % of Veh (*n* = 9–12). **(C)** Extracellular secretion of L-lactate from astrocytes after treatment with Veh (DMSO 0.1%) or GM1 (6–50 μM) for 1.5 and 3 h. Data are shown as the average +SEM of extracellular L-lactate (pmoles) expressed as % of Veh (*n* > 40). **(D)** Total protein in primary astrocytes during 1.5-and 3 h treatment with Veh (DMSO 0.1%) or GM1 (10 and 50 μM). Data are shown as the average +SEM of protein levels (pmoles/μg) expressed as % of Veh (*n* = 9–12). Statistical analyses consisted in one-way ANOVA followed by Dunnett’s post-hoc test; *p < 0.05, **p < 0.01, ***p < 0.001.

**FIGURE 2:**
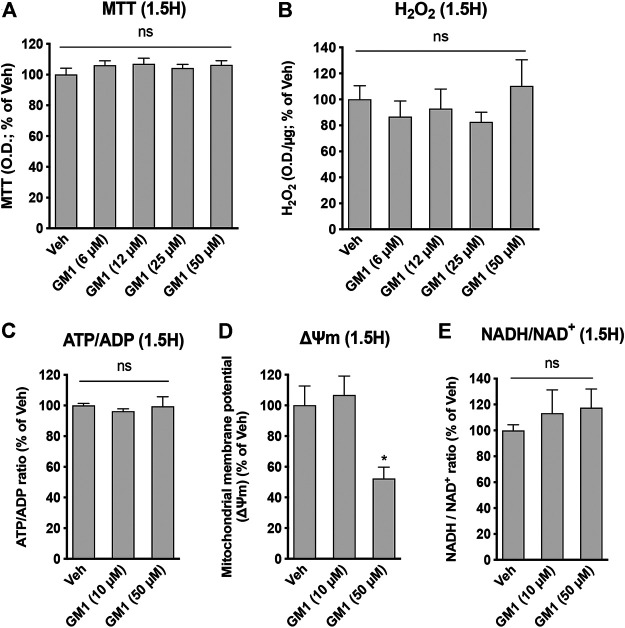
Modulation of astrocytic mitochondrial activity and redox state by GM1. **(A)** MTT assay used to monitor mitochondrial function through oxidoreductase enzymes activity in astrocytes treated for 1.5 h with Veh (DMSO 0.1%) or GM1 (6–50 μM). Data are shown as the average +SEM of optical density (OD) of mitochondrial reductase-induced formazan represented as % of Veh (*n* = 12). **(B)** Extracellular H_2_O_2_ secreted by astrocytes after treatment with Veh (DMSO 0.1%) or GM1 (6–50 μM) for 1.5 h. Data are shown as the average +SEM of H_2_O_2_ levels measured by OD (*n* = 7–10). **(C)** ATP/ADP ratio in astrocytes treated for 1.5 h with Veh (DMSO 0.1%) or GM1 (10 and 50 μM). Data are shown as the average +SEM of ATP/ADP ratio (*n* = 4). **(D)** Inner mitochondrial potential (ΔΨm) of astrocytes measured with JC1 dye after treatment with Veh (DMSO 0.1%) or GM1 (10 and 50 μM) for 1.5 h. Data are shown as the average +SEM of JC1 dye level (*n* = 6). **(E)** NADH/NAD^+^ redox ratio in astrocytes treated for 1.5 h with Veh (DMSO 0.1%) or GM1 (10 and 50 μM). Data are shown as the average +SEM of NADH/NAD^+^ redox ratio (*n* = 5–9). Statistical analyses consisted in one-way ANOVA followed by Dunnett’s post-hoc test; *p < 0.05, **p < 0.01, ***p < 0.001.

GM1 modulates glucose metabolism in astrocytes. GM1 is present in astrocytes and affects their morphology, growth and activity (Hefti et al., 1985; Skaper et al., 1986; Facci et al., 1987; Pyo et al., 1999; Min et al., 2004; Jou et al., 2006; Lee et al., 2012). Here, we investigated the effect of GM1 on astrocytic metabolism, and how this could impact neuronal protection. Astrocyte metabolism is mostly glycolytic and the secretion of lactate, one of the end products of glycolysis, is key for neuroprotection (Berthet et al., 2012; Jourdain et al., 2018), synaptic plasticity (Yang et al., 2014; Margineanu et al., 2018) and memory consolidation (Newman et al., 2011; Suzuki et al., 2011). First, our data indicate that GM1 stimulates glucose entry in astrocytes, as measured by 2-deoxyglucose (2DG) accumulation in the cells (Figure 1A). In line with this effect, glycogen, the storage form of glucose that is localized in astrocytes, is transiently decreased after treatment with GM1 at 1.5 h, while it is increased at 3 h time point in a concentration-dependent manner (Figure 1B). Next, treatment of astrocytes with GM1 was shown to trigger lactate secretion in a concentration-dependent manner, indicating increased glycolytic activity in astrocytes (Figure 1C). Data show no effect of GM1 on total protein levels at 1.5 or 3 h, indicating no sign of cellular toxicity (Figure 1D). To assess for the potential effect of GM1 on mitochondrial function, different functional parameters of mitochondrial activity were quantified after treatment with GM1. Results indicate that GM1 does not affect oxidoreductase enzymes activity as quantified by MTT assay at 1.5 h (Figure 2A), H_2_O_2_ secretion at 1.5 h (Figure 2B) and ATP/ADP ratio at 1.5 h (Figure 2C). However, at the highest concentration (50 μM), GM1 reduces astrocytic mitochondrial potential after 1.5 h of treatment (Figure 2D). Treatment of astrocytes with GM1 did not affect redox status as measured by NADH/NAD^+^ ratio (Figure 2E). Together, these data indicate that GM1 has a direct effect on astrocytes that leads to enhanced glycolysis activity, as shown by increased glucose uptake (Figure 1A), transient glycogen mobilization (Figure 1B) and lactate release (Figure 1C), while some parameters of functional mitochondrial activity such as inner mitochondrial potential (Figure 2D) were reduced at highest concentration tested. Thus, our data suggest that GM1 leads to rapid mobilization of glycogen through glycogenolysis (Figure 1B, left panel) to provide glucose source for glycolysis, leading to enhanced release of lactate by astrocytes (Figure 1C).

**FIGURE 3:**
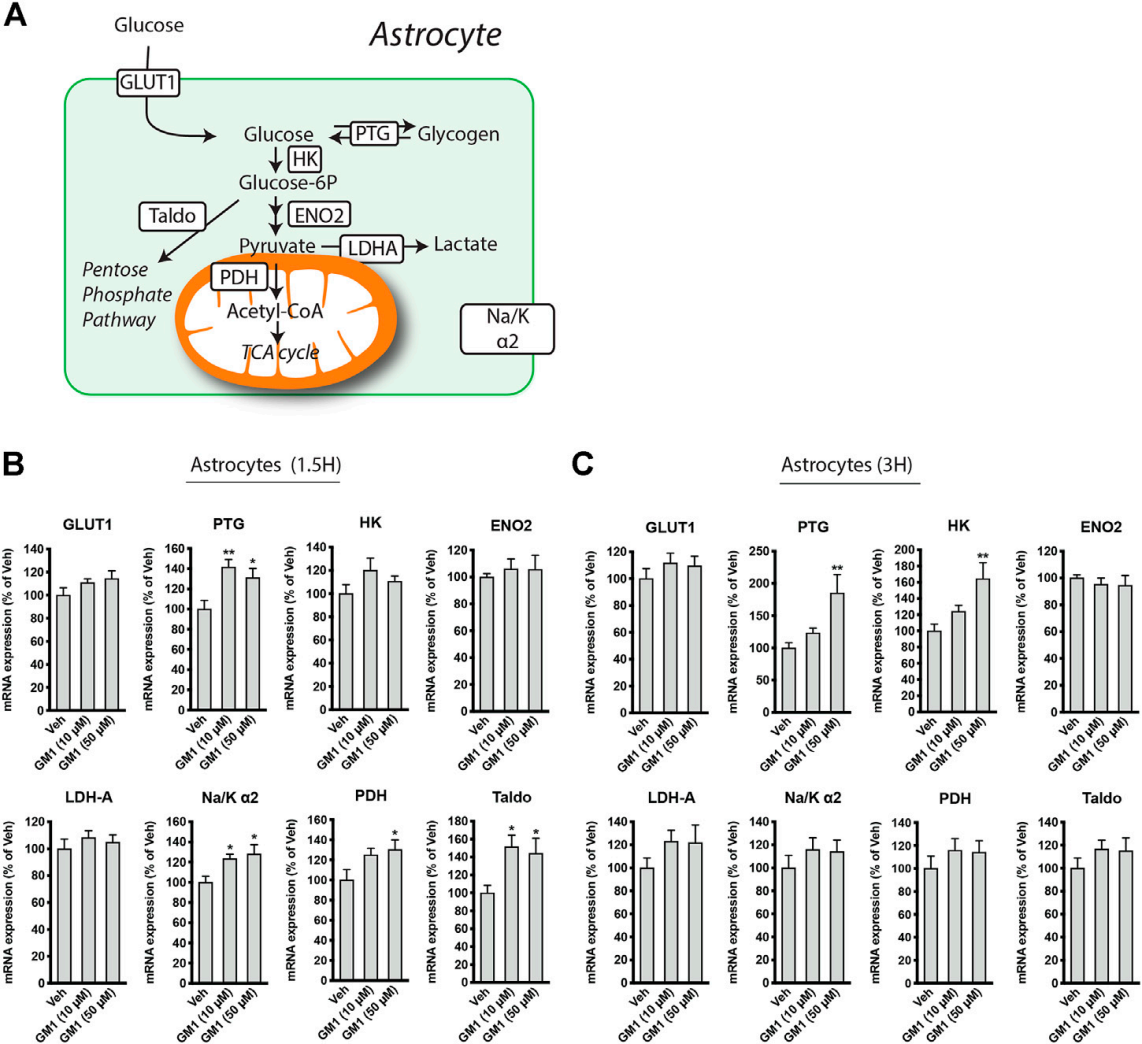
Regulation of astrocytic metabolic genes expression by GM1. **(A)** Scheme representing genes involved in astrocytic glucose metabolism pathways, including Glucose Transporter 1 (*Glut1*), Protein Targeting to Glycogen (*Ptg*), hexokinase (*HK*), Enolase 2 (*Eno2*), Lactate dehydrogenase A (*Ldha*), Na^+^/K^+^ ATPase subunit α2 (*Na/Kα2*), Pyruvate dehydrogenase (*Pdh*) and Transaldolase (*Taldo*). **(B,C)** Regulation of *Glut1*, *Ptg*, *HK*, *Eno2*, *Ldha*, *Na/Kα2*, *Pdh* and *Taldo* after treatment of astrocytes with Veh (DMSO 0.1%) or GM1 (10 and 50 μM) for 1 h **(B)** or 3 h **(C)**. Data are shown as the average +SEM of mRNA expression levels, represented as % of Veh (*n* = 12). Statistical analyses consisted in one-way ANOVA followed by Dunnett’s post-hoc test; *p < 0.05, **p < 0.01, ***p < 0.001.

GM1 stimulates the expression of metabolic genes in astrocytes. Because GM1 changes some aspects of astrocytic glucose metabolism, we next investigated its effect on the regulation of the expression of astrocytic genes involved in glucose metabolism. These genes include Glucose Transporter 1 (*Glut1*), the transporter of glucose expressed on astrocytes, Protein Targeting to Glycogen (*Ptg*), an enzyme that positively modulates glycogen synthesis, Hexokinase (*Hk*) and Enolase 2 (*Eno2*), two enzymes involved in glycolysis pathway, Transaldolase (*Taldo*), an enzyme of the pentose phosphate pathway, Pyruvate Dehydrogenase (*Pdh*), which catalyzes the conversion of pyruvate into acetyl-CoA, Na^+^/K^+^ ATPase subunit α2 (*Na/Kα2*), which is specifically expressed in astrocytes and restores Na^+^/K^+^ gradient after glutamate uptake in astrocytes, and Lactate Dehydrogenase A (*Ldha*), the enzyme that is responsible for the reduction of pyruvate into lactate (Figure 3A). Treatment of astrocytes with GM1 at 10 and 50 μM for 1.5 h (Figure 3A) and 3 h (Figure 3B) led to significant changes in the expression of a number of metabolic genes. Specifically, expression of *Ptg* at 1.5 and 3 h-time points was increased after treatment with GM1. Interestingly, the enhanced expression of the positive regulator of glycogen synthesis *Ptg* by GM1 (Figures 3B,C) could be linked to the increase of glycogen levels observed after 3 h treatment with GM1 (Figure 1B, right panel) that follows its rapid mobilization (Figure 1B, left panel).

Furthermore, gene expression of the glycolytic enzyme hexokinase was upregulated after treatment with GM1 at 3 h, while Na^+^/K^+^ ATPase subunit α2 was upregulated at 1.5 h. *Pdh,* which controls the conversion of pyruvate to acetyl-CoA and its entry into the TCA cycle, and *Taldo*, which is involved in the pentose phosphate pathway, respectively, were also transiently upregulated in astrocytes, indicating a general increase in glucose-mediated metabolic activity promoted by GM1.

**FIGURE 4:**
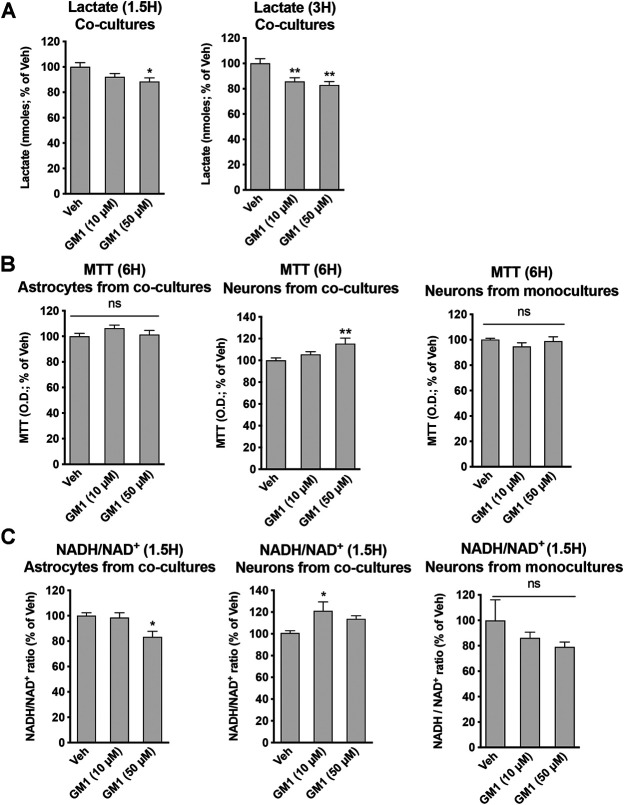
Modulation of glycolytic activity, mitochondrial activity and redox state by GM1 in neuron-astrocyte co-cultures. **(A)** Extracellular secretion of lactate from astrocyte-neuron co-cultures after treatment with Veh (DMSO 0.1%) or GM1 (10 and 50 μM) for 1.5 and 3 h. Data are shown as the average +SEM of extracellular lactate (nmoles) represented as % of Veh at 1.5 h (*n* = 12) **(B)** MTT assay used to monitor mitochondrial function in astrocytes **(left panel)** or neurons **(center panel)** from astrocyte-neuron co-cultures, or neurons from monocultures **(right panel)** treated with Veh (DMSO 0.1%) or GM1 (10 and 50 μM) for 6 h. Data are shown as the average +SEM of optical density of mitochondrial reductase-induced formazan, represented as % of Veh at 6 h (*n* = 11–12) **(C)** NADH/NAD^+^ redox ratio in astrocytes **(left panel)** or neurons **(center panel)** from astrocyte-neuron co-cultures, or neurons from monocultures **(right panel)** treated with Veh (DMSO 0.1%) or GM1 (10 and 50 μM) for 1.5 h. Data are shown as the average +SEM of NADH/NAD^+^ ratio, represented as % of Veh (*n* = 6). Statistical analyses consisted in one-way ANOVA followed by Dunnett’s post-hoc test; *p < 0.05, **p < 0.01, ***p < 0.001.

GM1 differentially modulates metabolism of astrocytes and neurons in co-cultures. To better understand the effect of GM1 on astrocytes and neurons, and the importance of the interplay between these cells, we next characterized the effect of GM1 in astrocyte-neuron co-cultures. Here, we analyzed metabolic parameters including extracellular lactate concentrations, mitochondrial activity and redox status in both cell types. Interestingly, the effect of GM1 in astrocyte-neuron co-culture on lactate secretion was different from that observed in astrocyte monocultures. While GM1 led to significant release of lactate in astrocyte monocultures, the treatment of astrocyte-neuron co-cultures led to a significant reduction in extracellular levels of lactate, which could be due to lactate consumption by neurons (Figure 4A). Mitochondrial activity of neurons in co-cultures was increased by GM1 in a dose dependent manner, while it did not affect mitochondrial activity of astrocytes in those co-cultures (Figure 4B). Interestingly, treatment of GM1 did not affect MTT levels in neurons from monocultures (Figure 4B, right panel), indicating that the presence of astrocytes is required to promote MTT increase in neurons. Redox status of astrocytes and neurons were also modulated in astrocyte-neuron co-cultures after treatment with GM1, as measured by NADH/NAD^+^ ratio (Figure 4C). Thus, in astrocytes from co-cultures, NADH/NAD^+^ ratio was decreased while in neurons from co-cultures, NADH/NAD^+^ ratio was increased after treatment with GM1. Conversion of pyruvate into lactate, which constitutes the final step of glycolysis, requires the conversion of co-factor NADH to NAD^+^. Hence, decreased NADH/NAD^+^ ratio in astrocytes from co-cultures triggered by GM1 indicates elevated glycolysis and utilization of NADH, while increased NADH/NAD^+^ ratio in neurons from co-cultures indicates lower glycolysis and favored oxidative phosphorylation (Figure 4C). Importantly, GM1 did not significantly affect NADH/NAD^+^ ratio in astrocytes from monocultures (Figure 2E), or in neurons from monocultures (Figure 3C, right panel), which indicates that the presence of astrocytes is required for the effect of GM1 on NADH/NAD^+^ ratio in neurons, and that the presence of neurons is required for the effect GM1 on NADH/NAD^+^ ratio in astrocytes.

**FIGURE 5:**
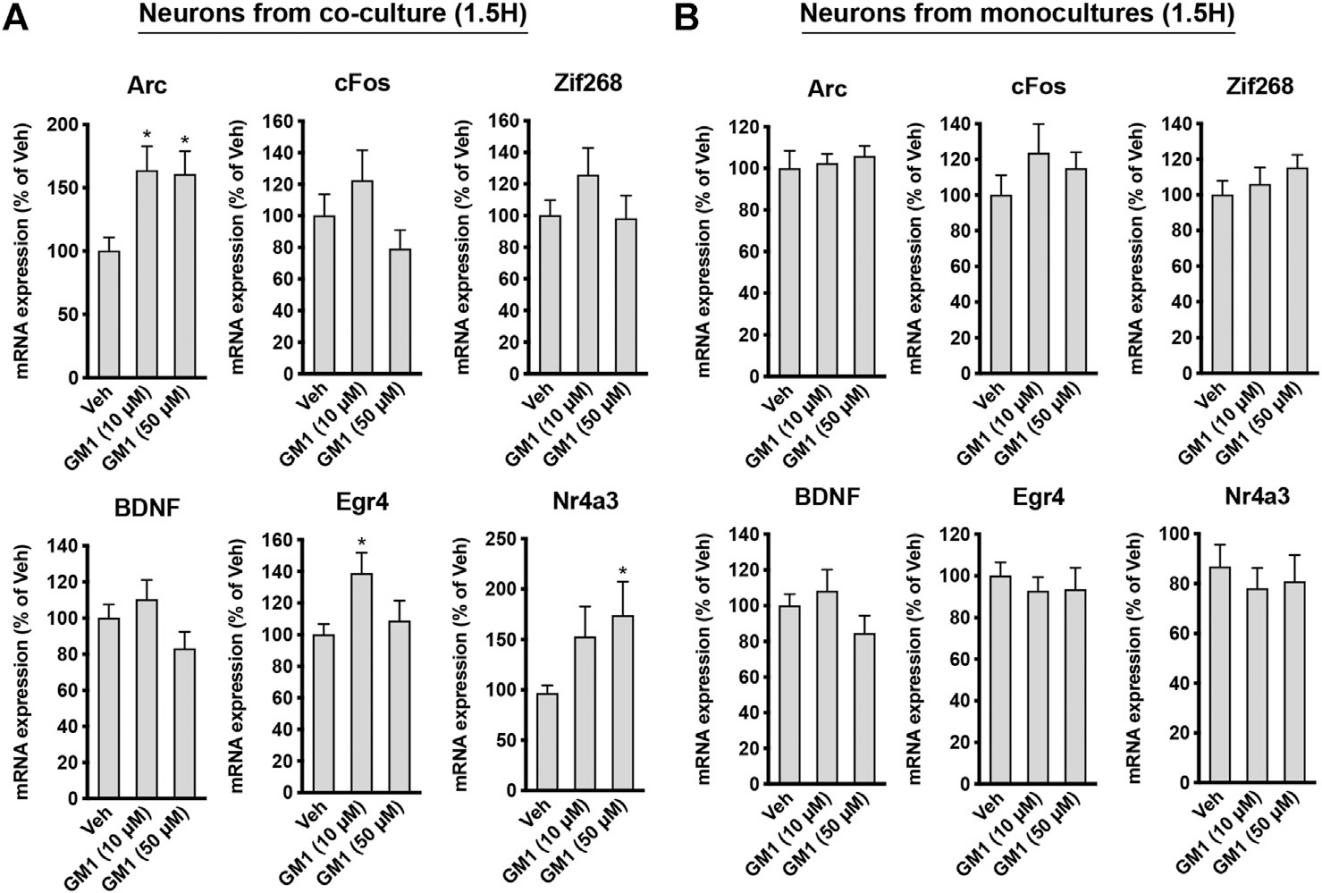
Regulation of plasticity and neuroprotection genes by GM1 in neurons from co-cultures and monocultures. **(A,B)** mRNA expression of *Arc*, *cFos*, *Zif268*, *Bdnf*, *Egr4* and *Nr4a3* in neurons from astrocyte-neuron co-cultures **(A)** or from monocultures **(B)** treated with Veh (DMSO 0.1%) or GM1 (10 and 50 μM) for 1.5 h. Data are shown as the average +SEM of mRNA expression levels represented as % of Veh (*n* = 12). Statistical analyses consisted in one-way ANOVA followed by Dunnett’s post-hoc test; *p < 0.05, **p < 0.01, ***p < 0.001.

**FIGURE 6:**
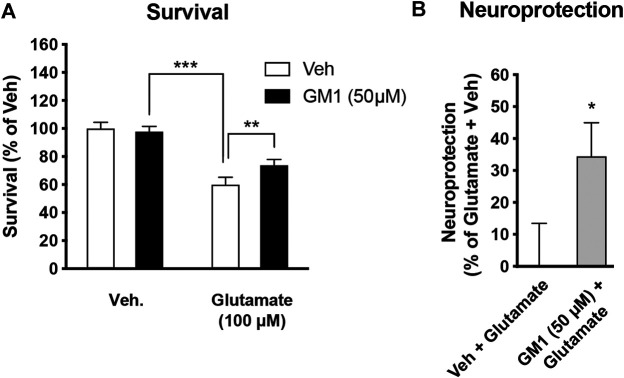
Neuroprotective effect of GM1 in astrocyte-neuron co-cultures. **(A)** Survival of neurons in astrocyte-neuron co-cultures was measured by MTT after exposure to medium (- glutamate) or glutamate (100 μM) for 10 min. Veh (0.1% DMSO) or GM1 (50 μM) was present in the medium 2 h before and 4 h after glutamate exposure, and MTT was used in neurons to monitor mitochondrial function and evaluate their survival. **(B)** Neuroprotective effect of GM1 was calculated as the percent (%) of neuroprotective effect of Veh (0.1% DMSO) vs. GM1 (50 μM); values of 0% (baseline) and 100% correspond to treatment without glutamate (+Veh) and with glutamate (+Veh), respectively. Data are shown as the average +SEM of MTT levels and represented as % of control values (*n* = 12). Statistical analyses consisted in one-way ANOVA followed by Bonferroni’s post-hoc test **(A)** or unpaired bilateral Student’s t test **(B)**; *p < 0.05, **p < 0.01, ***p < 0.001.

GM1 enhances the expression of neuroprotection genes in neurons from astrocyte-neuron co-cultures. Lactate is known to trigger the expression of a number of genes involved in neuroprotection and plasticity in neurons, including activity-regulated cytoskeleton-associated protein (*Arc*), *cFos*, *zif268* (also known as early growth response 1, *Egr1*), brain-derived neurotrophic factor (*Bdnf*), early growth response 4 (*Egr4*) and Nuclear receptor subfamily 4a group A member 3 (*Nr4a3*) (Yang et al., 2013; Margineanu et al., 2018). Because GM1 modulates astrocytic glucose metabolism and triggers lactate release, we next assessed the effect of GM1 on the expression of lactate-induced neuroprotection genes in neurons from astrocyte-neuron co-cultures (Figure 5A) and from monoculture (Figure 5B). The expression of several genes including *Arc*, *Egr4* and *Nr4a3* was induced by GM1 in neurons when they were cultured in the presence of astrocytes (Figure 5A), while their expression did not change in neuron monocultures (Figure 5B). These data indicate that GM1 modulates the expression of a number of neuroprotection genes through an effect that requires the presence of astrocytes. Finally, we showed that treatment of astrocyte-neuron co-cultures with GM1 had neuroprotective effects in cultures exposed to an excitotoxic concentration of glutamate (100 μM). Thus, while exposure of astrocyte-neuron co-cultures to glutamate led to significant neuronal mortality, as measured by MTT assay, treatment of the co-cultures with GM1 led to significant reduction of glutamate-induced excitotoxic effect (Figure 6).

## Discussion

**FIGURE 7:**
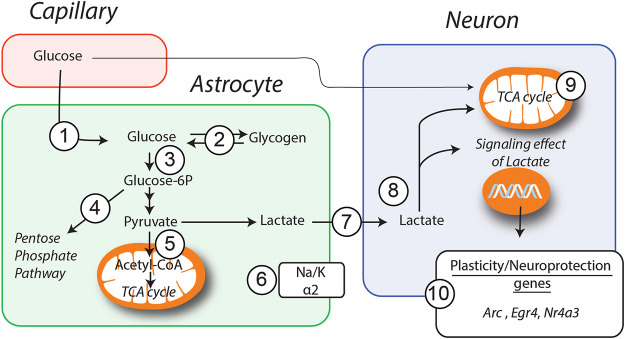
Schematic representation of GM1 metabolic effects in astrocytes and neurons. Models of how GM1 induces glucose entry (1) and glycogen mobilization (2) in astrocytes, leads to the upregulation of *Ptg* (2), *Hk* (3), *Taldo* (4), *Pdh* (5) and *Na* ^*+*^ */K* ^*+*^ *atpase subunit α2* (6) genes, enhances secretion of lactate (7) and its transfer to neurons (8). When neurons are cultured in presence of astrocytes, treatment with GM1 leads to increased neuronal mitochondrial activity (9) and expression of a number of genes including *Arc*, *Egr4* and *Nr4a3* (10).

A number of evidence *in vitro*, *in vivo* and in human studies indicate that GM1 has neuroprotective effects in a variety of neurological disorders. However, the mechanisms underlying positive effects of GM1 remain unclear. As summarized in Figure 7, we show here that GM1 enhances glucose uptake, mobilizes glycogen stores, and stimulates secretion of lactate by astrocytes, which is accompanied by the enhanced gene expression of a number of metabolic enzymes including PTG, Hexokinase, Na^+^/K^+^ ATPase subunit α2, Pyruvate Dehydrogenase and transaldolase. In line with these results, GM1 modulates NADH/NAD^+^ ratio in astrocyte-neuron co-cultures, in a manner consistent with enhanced glycolysis in astrocytes and oxidative phosphorylation in neurons. Finally, our data indicate a neuroprotective effect of GM1 in astrocyte-neuron co-cultures, while it mediated astrocyte-dependent expression of a number of neuroprotection genes in neurons.

Energy metabolism is tightly regulated in the brain, and astrocytes play a fundamental role in neurometabolic coupling. Astrocytes, which are highly glycolytic, provide neurons with energy sources including lactate, one of the metabolic end-products of glycolysis. Aerobic glycolysis in astrocytes is triggered by glutamate uptake, which then leads to transfer of lactate from astrocytes to neurons, known as the astrocyte-neuron lactate shuttle (Pellerin and Magistretti, 2012; Magistretti and Allaman, 2018). In addition to its important role as an energy substrate, lactate acts as a signaling molecule in the brain, modifying neuronal excitability (Sada et al., 2015), promoting neuroprotection (Berthet et al., 2012; Jourdain et al., 2018) and enhancing the expression of synaptic plasticity genes and memory (Newman et al., 2011; Suzuki et al., 2011; Yang et al., 2014; Margineanu et al., 2018; Harris et al., 2019).

Here, we show that GM1 enhances glycolysis in astrocytes, leading to glucose uptake and release of lactate (Figures 1A–C). Glycogen is the main source of glucose storage in the brain, located in astrocytes. Astrocytic glycogen mobilization is triggered by neurotransmitters including vasoactive intestinal peptide (VIP) and noradrenaline (Magistretti et al., 1981). We show that GM1 promoted glycogenolysis in astrocytes over a short time period (1.5 h), while it led to resynthesis of glycogen at a later time point (3 h), indicating a dynamic effect of GM1 on astrocytic glycogen stores (Figure 1B). Additional data showed that, while GM1 decreased astrocytic mitochondrial inner membrane potential at highest concentration tested (i.e. 50 μM), it did not affect other mitochondrial functional parameters in astrocytes such as oxidoreductase enzymes activity, ATP production, H_2_O_2_ release, or redox status changes (Figure 2). Next, we found that GM1 enhances the expression of a number of metabolic genes in astrocytes (Figure 3). GM1 triggers the expression of *Ptg*, the glycogen-binding subunit of protein phosphatase-1 (PPP1) that positively regulates glycogen synthesis through activation of glycogen synthase and inactivation of glycogen phosphorylase (Greenberg et al., 2003). Thus, increase of *Ptg* is in line with metabolic adaptation in astrocytes leading to storage of glycogen observed after 3 h-treatment with GM1. GM1 also led to the increased expression of hexokinase, which phosphorylates glucose into glucose-6-phosphate as the first step of glucose entry in the cell, and pyruvate dehydrogenase, the enzyme that catalyzes the transformation of pyruvate into Acetyl-CoA. GM1 also enhances the expression of the α2 subunit of the Na^+^/K^+^ ATPase, which restores sodium/potassium gradient in astrocytes and was shown to be upregulated in response to glutamate uptake (Brunet et al., 2004). Finally, expression of transaldolase is increased by GM1, indicating enhanced glucose entry into the pentose phosphate pathway, the main producer of reducing equivalents NADPH used to regenerate glutathione that scavenges reactive oxygen species (ROS) (Wamelink et al., 2008). Together, these data indicate that GM1 triggers the expression of a number of genes in astrocytes that control different aspects of glucose metabolism including aerobic glycolysis, eventually leading to glucose uptake, glycogen mobilization and lactate release.

In astrocyte-neuron co-cultures, treatment with GM1 led to the reduction of extracellular lactate levels, in contrast to the lactate release-enhancing effects of GM1 in astrocytes monocultures (Figure 4A). This indicates a different outcome for GM1 on lactate release if neurons are present or not, most likely through the utilization of lactate by neurons. In line with this observation, treatment of astrocyte-neuron co-cultures with GM1 led to enhanced mitochondrial activity in neurons, as measured by oxidoreductase enzymes activity, while it did not affect mitochondrial activity in astrocytes (Figure 4B). Furthermore, treatment of neuron monocultures with GM1 did not lead to any changes in mitochondrial activity, indicating that GM1-mediated effect on neuronal mitochondrial activity requires the presence of astrocytes. Redox status of astrocytes and neurons was modulated in co-cultures after treatment with GM1, as shown by NADH/NAD^+^ ratio (Figure 4C). Thus, in astrocytes from co-cultures, NADH/NAD^+^ ratio was decreased after treatment with GM1, indicating the oxidation of cofactor NADH to NAD^+^ in the glycolytic process of pyruvate to lactate conversion. In contrast, NADH/NAD^+^ ratio was increased in neurons from co-cultures treated with GM1, which underlines the conversion of lactate into pyruvate. In contrast, GM1 treatment of neuron monocultures did not lead to any significant changes in NADH/NAD^+^ ratio, indicating that GM1-mediated effect on neuronal NADH/NAD^+^ ratio requires the presence of astrocytes. Together, these data indicate that GM1 modulates astrocytes and neurons in a different fashion when in co-culture, by enhancing glycolysis in astrocytes and oxidative phosphorylation in neurons.

Lactate not only plays a role as an energy substrate, but also mediates the expression of a number of synaptic plasticity and neuroprotection genes through NMDA receptor-dependent mechanisms (Margineanu et al., 2018; Yang et al., 2014). Because GM1 triggers the secretion of lactate by astrocytes and stimulates oxidative phosphorylation in neurons when co-cultured with astrocytes, we next investigated the effect of GM1 on the expression of these genes in neurons, when cultured alone or in the presence of astrocytes. Our data indicate that GM1 triggers the expression of *Arc*, *Egr4* and *Nr4a3* in neurons only when neurons are cultured in the presence of astrocytes (Figure 5A). In contrast, expression of these genes was not changed in neuronal monocultures, indicating that the effect of GM1 only occurs when astrocytes are present. Consistently, other data showed that GM1 activates the mitogen-activated protein kinase (MAPK) pathway in complex brain preparations such as cortical prisms, as well as in mixed cultures only when astrocytes were present (Fiumelli et al., 2016). Finally, GM1 displays a neuroprotective effect in a model of glutamate-mediated excitotoxicity in astrocyte-neuron co-cultures (Figure 6). This observation is in line with the neuroprotective effect of GM1 that has been described in several neuropathologies including SCI, HD, and PD. These pathologies, along with other prevalent neurodegenerative diseases such as ALS and AD, exhibit brain energy metabolism impairments and involvement in astrocytic dysfunction (Vandoorne et al., 2018; Butterfield and Halliwell, 2019). Targeting astrocytes to restore brain energy metabolism impairments, potentially through GM1-mediated actions, may constitute new therapeutic strategies for hypometabolic neuropathologies (Finsterwald et al., 2015).

Several clinical studies indicate that GM1 has beneficial effects in SCI (Geisler et al., 2001; Geisler et al., 1991). Glial cells including oligodendrocytes and Schwann cells are metabolically coupled to axons and provide energy substrates to regulate axonal bioenergetics and integrity (Lee et al., 2012; Saab et al., 2016; Jha et al., 2020). A recent study showed that SCI lesion leads to adaptation through increased glycolytic flux and lactate extrusion by Schwann cells, which is accompanied by the expression of glycolytic genes through mTOR-dependent mechanisms (Babetto et al., 2020). Suppression of pyruvate or lactate production in Schwann cells through inhibition of Glucose Transporter 1 (GLUT1) or Lactate Dehydrogenase A (LDHA) accelerated axonal degeneration in injured tissue, while improving glycolysis by targeting mTOR alleviated axonopathy (Babetto et al., 2020). Another study recently showed that enhancing glycolysis in glia promoted morphological and functional recovery after CNS injury through an effect mediated, at least in part, by lactate (Li et al., 2020).

A number of evidences also point to the positive effect of GM1 in HD, which is characterized by brain glucose hypometabolism in the striatum and cerebral cortex early in the genesis of the pathology. Glucose uptake, ATP generation, aerobic glycolysis and mitochondrial function are all impaired in HD (Liot et al., 2017; Morea et al., 2017; Illarioshkin et al., 2018). PET measurements indicate that hypometabolism is mostly caused by decreased glycolysis in HD (Powers et al., 2017). Importantly, several astrocytic functions that regulate ion homeostasis, calcium signaling, neurotransmitter clearance and energy metabolism were found to be impaired in HD (Khakh et al., 2017; Skotte et al., 2018; Wilton and Stevens, 2020). Synthesis of gangliosides is decreased in cellular and animal models of HD, as well as in fibroblasts from patients (Denny et al., 2010; Maglione et al., 2010). *In vitro*, GM1 was found to have neuroprotective effect in striatal cells expressing Huntingtin (Maglione et al., 2010). In different transgenic mouse models of HD including R6/2, Q140 and YAC128 mice, motor function was improved after GM1 administration (Di Pardo et al., 2012; Alpaugh et al., 2017). GM1 also ameliorated anxiety-like and depression-like behaviors, and improved cognitive functions in Q140 and YAC128 HD mouse models (Alpaugh et al., 2017).

PD also has clear links with energy metabolism due to a large degree to disease-associated mitochondrial defects (Anandhan et al., 2017). Recent evidence indicates that downregulation of astrocyte-specific genes such as Excitatory Amino Acid Transporter 2 (*EAAT-2*) and Aquaporin 4 (*AQP4*) is associated with PD (Zhang et al., 2016; Prydz et al., 2017). Further, induced pluripotent stem cells (iPSC)-derived astrocytes from PD patients were found to contribute to non-cell autonomous neurodegeneration (di Domenico et al.,et al., 2019). Decrease in the expression of the glycolytic enzyme phosphoglycerate kinase 1 (PGK1) is also associated with PD (Sakaue et al., 2017). In patients, the use of ^18^F-Fluorodeoxyglucose (FDG) PET allows the identification of regional glucose hypometabolism and is increasingly recognized as a tool for PD diagnosis (Meyer et al., 2017; Albrecht et al., 2019). GM1 was shown to rescue damaged dopaminergic neurons, increase striatal dopamine levels and reduce loss of neurons in substantia nigra in mice and monkeys treated with the neurotoxin 1-methyl-4-phenyl-1,2,3,6-tetrahydropyridine (MPTP) (Schneider and Yuwiler, 1989; Stull et al., 1994). Clinical studies indicated significant improvement in PD patients treated with GM1 (Schneider et al., 1995; Schneider et al., 1998; Schneider et al., 2013).

Given that GM1 enhances astrocytic glycolysis, which leads to increased glucose uptake and lactate secretion, the use of GM1 may be beneficial in pathologies that exhibit hypometabolism and reduced glycolysis. In this context, the effects of GM1 mediated by astrocytes that leads to enhanced neuronal mitochondrial activity and expression of neuroprotection genes could explain, at least in part, the positive therapeutic outcome of GM1 in neuropathologies such as SCI, HD and PD. A better understanding of the molecular mechanisms underlying the effects of GM1 that lead to astrocytic glycolysis will be key to assess for potential novel efficient ganglioside-based therapies for hypometabolic neuropathologies.

## Data Availability

The original contributions presented in the study are included in the article/Supplementary Material, further inquiries can be directed to the corresponding author.
